# Prognosis factors in the treatment of bisphosphonate-related osteonecrosis of the jaw - Prognostic factors in the treatment of BRONJ

**DOI:** 10.4317/jced.51213

**Published:** 2014-02-01

**Authors:** Daigo Yoshiga, Ikuo Nakamichi, Yoshihiro Yamashita, Noriaki Yamamoto, Kensuke Yamauchi, Shinnosuke Nogami, Takeshi Kaneuji, Sho Mitsugi, Kenkou Tanaka, Yoshihiro Kataoka, Takuma Sakurai, Hiroyasu Kiyomiya, Ikuya Miyamoto, Tetsu Takahashi

**Affiliations:** 1 Division of Oral and Maxillofacial Reconstructive Surgery, Department of Oral and Maxillofacial Surgery, Kyushu Dental University, Fukuoka, Japan; 2 Division of General Internal Medicine, Department of Health Promotion, Kyushu Dental University, Fukuoka, Japan; 3 Section of Oral Surgery, Department of Oral and Maxillofacial Surgery, Fukuoka Dental College, Fukuoka, Japan; 4 Department of Oral and Maxilla-facial Surgery, Faculty of Medicine, Oita University, Oita, Japan; 5 Division of Oral and Maxillofacial Surgery, Department of Oral Medicine and Surgery, Tohoku University Graduate School of Dentistry, Sendai, Japan

## Abstract

Objectives: Bisphosphonate-related osteonecrosis of the jaw (BRONJ) is a relatively rare but serious side effect of bisphosphonate (BP)-based treatments. This retrospective study aimed to investigate the risk factors and predictive markers in cases where patients were refractory to a recommended conservative treatment offered in our hospital. Patients and Methods: This single-center study collated the medical records of all patients treated for BRONJ between 2004 and 2011. A complete medical history, including detailed questionnaires, was collected for all patients, focusing on identifying underlying risk factors, clinical features, location and bone marker levels of BRONJ. Results: The mean BRONJ remission rate was 57.6%, and the median duration of remission was seven months. Eighteen patients (34.6%) had persistent or progressive disease with a recommended conservative treatment for BRONJ. Notably, urinary cross-linked N-terminal telopeptide of type 1 collagen (NTX) levels in those resistant to conservative treatment tended to be lower than in patients that healed well. Conclusions: We confirm that a significant proportion of BRONJ sufferers are refractory to a recommended conservative treatment and find that anticancer drugs, periodontal disease, the level of bone exposure and the dosage of intravenous BPs (e.g. zoledronate) represent specific risk factors in BRONJ that may determine the success of a recommended conservative treatment. Additionally, the NTX levels might be able to be a prognostic factor for the conservative treatment of BRONJ; additional research is necessary.

** Key words:**Bisphosphonate, osteonecrosis, jaw, prognostic, retrospective.

## Introduction

Bisphosphonates (BPs) are a class of drugs derived from pyrophosphates, which are endogenous inorganic regulators of mineralization. The substitution of oxygen atoms in the basic pyrophosphate chain with carbon inhibits osteoclasts, causing reduced bone resorption (1,2). BPs are very effective in the management of malignancy-related hypercalcemia and skeletal events associated with multiple myeloma, bone metastasis from breast cancer and prostate cancer, and osteoporosis (3). However, nitrogen-containing BPs are also associated with an increased risk of developing osteonecrosis of the jaw; zoledronate and pamidronate, drugs used commonly to stabilize metastatic cancer deposits in the bone, appear to be particularly closely linked to this phenomenon (4). Many cases of bisphosphonate-related osteonecrosis of the jaw (BRONJ) have been reported globally and, although the incidence of BRONJ is generally thought to be low, its occurrence impacts negatively on oral hygiene and patient quality of life (QOL). BRONJ is extremely intractable because neither the pathogenic mechanism nor a standard therapy for BRONJ has been established. Conventional therapy bears similarity to that for osteomyelitis of the jaw, involving active surgical resection (e.g. sequestrectomy) and hyperbaric oxygen treatment (5). However, the American Association of Oral and Maxillofacial Surgeons (AAOMS) and the Japanese Society of Oral and Maxillofacial Surgeons (JSOMS) now recommend a more conservative treatment strategy (1,6), such as the use of antibiotics, gargling with antimicrobial agents, and local irrigation and surgical debridement, while there are some cases that are resistant to these treatment. Additionally, BRONJ risk fctors were categorised as drug-related, local and demographic or systemic factors (1). Other medications, such as steroids, thalidomide and other Anti-cancer drugs, were thought to be risk factors, however no measurable associations were identified. We therefore performed a clinical analysis of the risk factors evident in intractable BRONJ cases following a recommended conservative treatment in our department.

## Patient and Methods

Between April 2004 and May 2011, 52 patients (8 males, 44 females) were referred to our hospital for prevention or treatment of BRONJ. Our protocol adhered strictly to AAOMS guidelines, which necessitate the identification of the following: 1) Exposed bone in the maxillofacial region over a period of 8 weeks; 2) current or previous treatment using BPs; and 3) no prior history of radiation therapy to the jaw region.

The treatment objectives for patients with an established diagnosis of BRONJ are to eliminate pain, control infection of the soft and hard tissue, and minimize the progression or occurrence of osteonecrosis. The staging of BRONJ was performed according to AAOMS 2009 recommendations, which suggest that surgical debridement and/or a resection approach are indicated only in patients with advanced stage disease (either stage 3, or stage 2 disease that is resistant to antibiotics). We retrospectively reviewed the age, gender, affected site, BRONJ staging, type of pharmaceutical BP preparation, reason for taking BPs, duration of BP use, method of administration, presence of withdrawal, bone denudation areas, risk factors (namely: diabetes mellitus, steroids, anticancer drugs, smoking, drinking, and periodontal disease), history of dental surgical treatment related to BP medication and any previous treatment for BRONJ based on clinical records, intraoral photography, and radiological assessment at the initial diagnosis. We selected cases (stages 0–3) that were treated conservatively, including by instruction in oral hygiene, administration of antibiotics, antimicrobial mouth rinses, local irrigation, and sequestrectomy according to the BRONJ guidelines defined by the AAOMS and the JSOMS. The cure period was defined as the time taken for mucosa to completely cover necrotic tissue and exposed bone. Furthermore, we investigated the cure period for patients who were initially diagnosed as stages 1–3 with bone exposure using the Kaplan–Meier method. The levels of urinary cross-linked N-terminal telopeptide of type 1 collagen (NTX) were measured during a periodic check-up in osteoporosis management after BRONJ diagnosis. A group of 13 patients on BP therapy for osteoporosis for >6 months but with no occurrence of BRONJ were used as a control group for the NTX measurements, and were also monitored during a periodic check-up for osteoporosis management.

Statistically significant differences were tested using the Mann-Whitney-U test.

## Results

**Figure 1:**
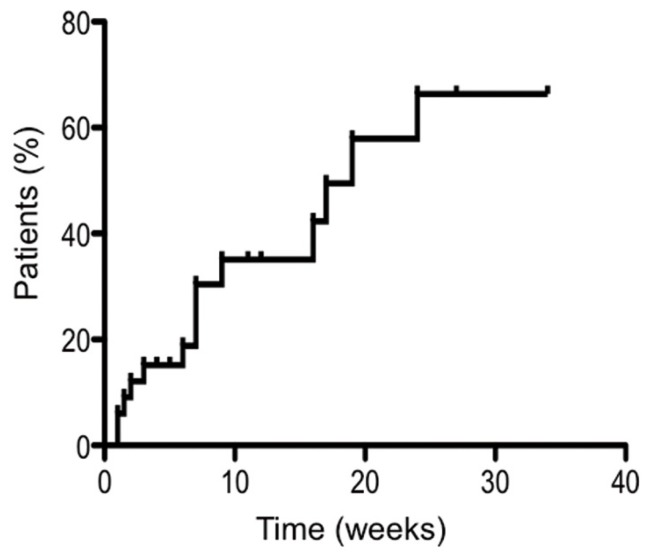
Kaplan-Meier curve showing the time after diagnosis until no further bone exposure was evident.

The 52 patients selected were all referred to our hospital for intraoral symptoms such as swelling, pain, pus discharge, and bone denudation. Of these, 19 cases (36.5%) were stage 0, five cases (9.6%) were stage 1, 22 cases (42.3%) were stage 2 and six cases (11.5%) were stage 3. When the stage distribution was analyzed according to age, we found the greatest proportion of patients to be in their 70’s (19 patients). This was similar to the distribution of patients treated with BPs, as in previous reports. Stage 3 disease was present only in patients over 60 years of age. Furthermore, we investigated the cure period for patients who were initially diagnosed as stages 1–3 with bone exposure using the Kaplan–Meier method. In this analysis, the cure period was defined as ending at the point when no bone exposure remained. In terms of the therapeutic ratio, there were no major differences between women and men. The cure period ranged from 1–24 months, and the median cure period was seven months. Of the 33 patients included in this group, seven (21.2%) either died or dropped out during the treatment period and the cure rate of the remainder was 61.5% (16 patients) (Fig. 1). Therefore, we investigated intractable cases for which the cure period was longer than 7 months or in which the disease progressed to a higher stage during conservative treatment.

According to stage distribution, many intractable cases were classified as stage 2. Of the intractable cases, eight (44%) developed BRONJ following tooth extraction, and seven of these developed BRONJ due to tooth extraction without withdrawal of BPs. The single remaining case (1.9%) developed BRONJ following tooth extraction 3 months after withdrawal of alendronate. The affected site was most often (66.7%) in the mandible in patients with stage 0–3 disease. In the intractable cases, the incidence of lesions in the maxilla, mandible and both jaws was 16.7%, 72.2% and 11.2%, respectively.

BPs were administered by injection in 23% of stage 0–3 cases and in 50% of intractable cases. In all stage 0–3 cases, the BP administered was a second-generation drug (alendronate) in 50% of cases, whereas the remainder were treated with third-generation drugs in the order of frequency risedronate>zoledronate>minodronate. Of the patients that were refractory to conservative treatment, 44% were treated using zoledronate, whereas for alendronate this figure was 33%. Additionally, 80% of patients administered zoledronate were found to be refractory to conservative treatment.

Among all BRONJ cases (stage 0–3), the reason for administering BPs was osteoporosis in 39 patients (75%), treatment of bone metastasis in 21% (breast cancer: 7 patients, thyroid cancer: 1 patient, stomach cancer: 2 patients, rectal cancer: 1 patient), and other reasons in two patients (4%). Of the refractory cases, those administered BPs for cancer bone metastases included nine patients that accounted for half of those resistant to conservative treatment. Additionally, in six patients (11.5%), BPs were combined with anticancer drugs. Five of the nine cancer patients died of other illnesses during the course of BRONJ treatment. Furthermore, we investigated the presence of other risk factors for BRONJ; diabetes mellitus was observed in four refractory patients (22.2%), but no diabetic patients were found among the resolved cases. Steroid therapy in particular has been reported to have negative effects on bone tissue (7), but when we analyzed patients treated using BPs in combination with corticosteroids, three patients showed complete resolution of the disease. As for smoking and drinking, there were no significant differences between refractory and resolved cases. Among the refractory cases, 66.6% showed poor oral hygiene, including deposition of plaque and the presence of periodontal disease. The period of BP treatment for all cases ranged from 1–130 months (stage 0–3), with a mean of 46.4 months. The mean duration of BP ad-ministration until onset of BRONJ was 41.6 months (7–70 months) in resistant cases; however, there were no significant differences compared with patients showing good healing. BPs were withdrawn from one patient (3%) exhibiting good healing, whereas BPs were not withdrawn from seven refractory patients (38.9%). In these seven patients, we preferred to use BPs for treatment of cancer bone metastases under consultation with a physician.

Furthermore, we investigated and evaluated the range of bone exposure area during our initial consultation. We evaluated the area of bone exposure according to the number of teeth, using X-rays, medical records, and intraoral photographs. Refractory patients showed a significantly wider bone exposure range compared with patients in whom healing was good. The average bone exposure range was 2.6 teeth across all patients, 1.6 teeth (range 1–2) for those showing good healing, and 3.4 teeth (range 1–7) for refractory patients ( Table 1).

**Table 1:**
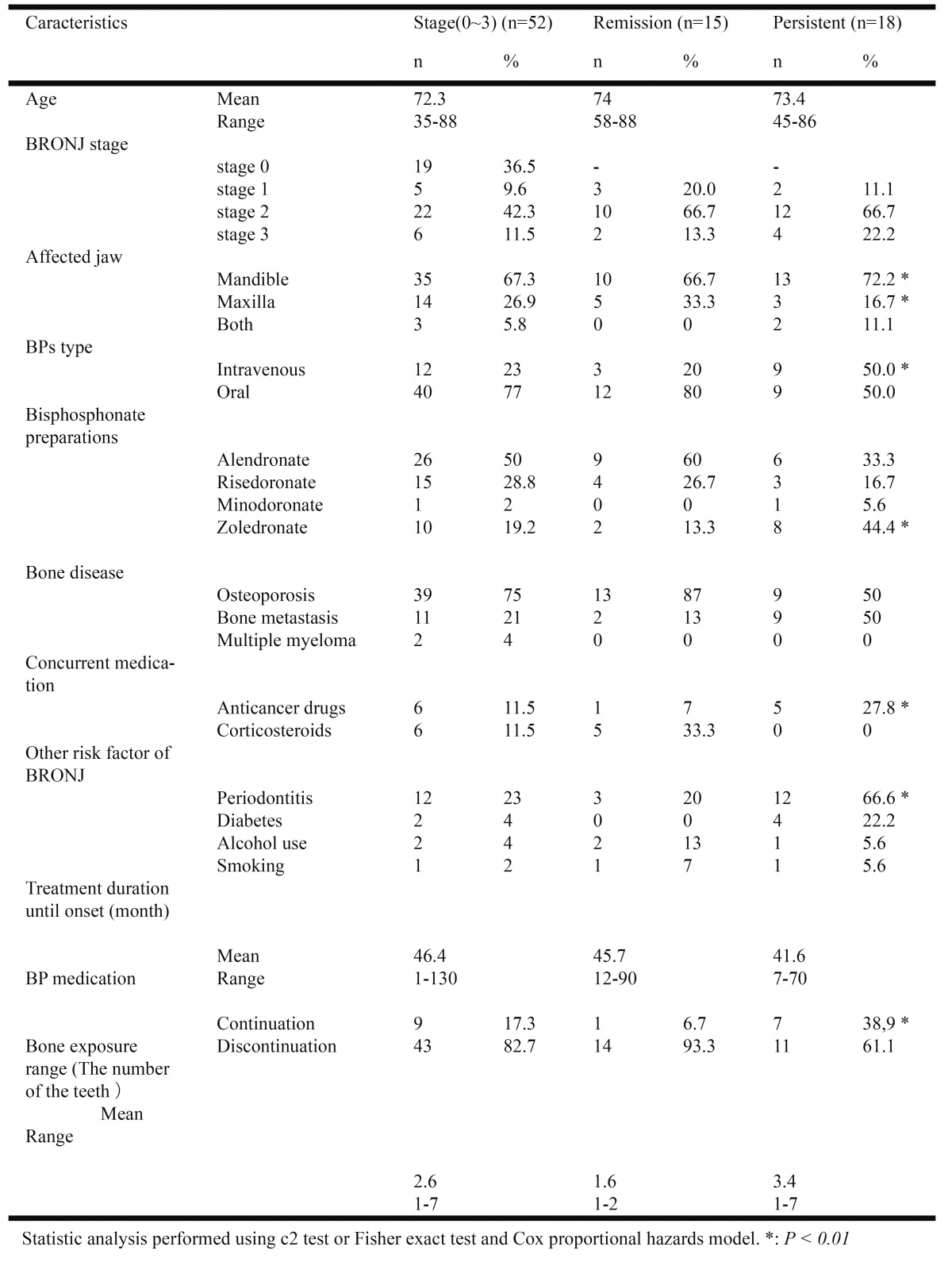
Statistical analysis results between patient characteristics and outcome of the conservative treatment.

**Figure 2:**
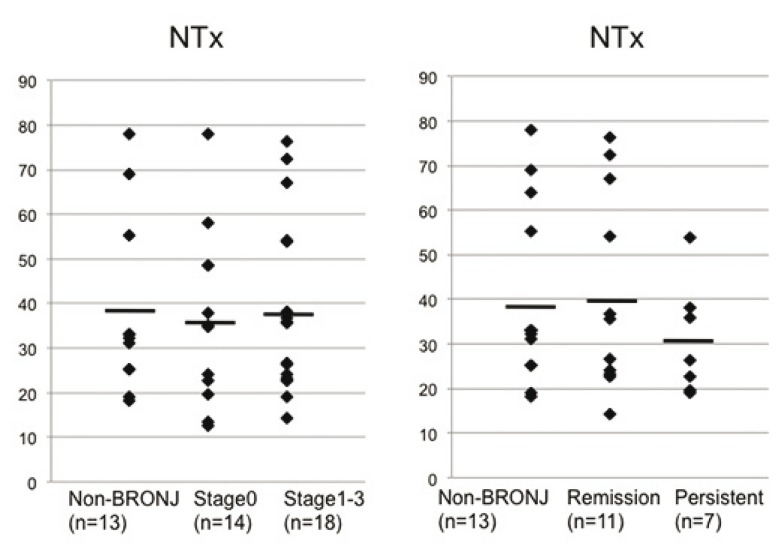
Urinary levels of bone resorption marker cross-linked amino-terminal telopeptide of type I collogen (NTX). The mean of the Persistent group was lower than that of the control and remission group but there was no significance (P = 0.11).

Data on urinary NTX levels were collected from the physicians that had initially prescribed the BP drugs. Generally, daily variation of the urinary marker is large and is inferior in plasticity, whereas the measurement error is small and the serologic marker is superior in plasticity. The urinary NTX levels in the treatment-refractory group were low (19.2–54 nmol BCE/mmol Cr (mean: 30.8 ± 12.6)) compared with the control (mean: 39.7 ± 19.7) and remission (mean: 41.1 ± 22.3) groups, although these differences were not significant (P = 0.11). Our data showed that there were no significant differences in NTX levels between patients with no BRONJ or BRONJ stage 0 and those with stage 1–3 disease, although NTX levels tended to be lower in those patients refractory to conservative treatment than in patients showing good healing (Fig. 2).

## Discussion

BRONJ is a recognized side effect of treatments using BPs and involves osteonecrosis of the jaw caused by disordered wound healing after invasive dental treatment such as tooth extraction, periodontal surgery, endodontic treatment, or periodontal treatment. Currently, there is no definitive preventative method and no universally recommended treatment modality. Since osteonecrosis of the jaw was first reported as a serious side effect of BP treatment by Marx (4) in the United States in 2003, similar findings have been reported elsewhere (1,2). In Europe and the United States, more than 2,500 cases of BRONJ have been reported (8), with the majority being in patients receiving BP injections to treat cancer, and a study by the AAOMS showed that 94% of 364 BRONJ patients received such injections, whereas BRONJ caused by oral administration accounted for only 4.7% (1). However, the results of a nationwide survey by the JSOMS in 2007 (1) indicated that injections accounted for only 73.3% of BRONJ cases whereas disease arising following oral administration was much more common in Japan than in the United States. In our study, 23% of patients with stage 0–3 disease received injections at stage 1 or higher, and exposed bone was observed in 12 patients (33%) treated using injections. Regarding oral medication, many more cases of BRONJ induced by alendronate were observed than reported in previous studies (1,9,10). In our study, BPs were administered by injection in 50% of refractory patients. In Japan, a potentially greater number of BRONJ patients are treated using oral medication than reported in the United States. According to an investigation by JSOMS, cases of BRONJ developing following BP injection have been reported to be more severe than those following oral administration (3). Our survey found more BRONJ cases induced by oral medication than previous reports, while half of the refractory cases were induced by BP injection and tended to respond poorly to conservative treatment. More women (44 patients, 85%) than men (8 patients, 15%) tended to suffer from BRONJ, as expected given the indications for BP treatment. Furthermore, we found most cases to be in patients over 60 years of age, so a high percentage of senior citizens were recognized in our study population, consistent with a report by the American Dental Association (1).

Consistent with previous studies, we found that BRONJ most commonly affects the lower jaw. This is because the lower jaw, like other bones such as the femur, is surrounded by cortical bone, but the teeth anchored in this bone extend into the bone marrow from the oral cavity providing periodontal microorganisms a path of ingress to that site. Furthermore, the oral mucosa is thin and susceptible to injury caused by, for example, dentures. Bone metabolism in alveolar bone is estimated to be higher than that of long bones (~10 times that of the tibia and 3–5 times that of the inferior border of the mandible) making it more susceptible to agents affecting remodeling (11). Therefore, a large uptake of BPs coupled with an enhanced environment for further bone metabolism facilitates the development of BRONJ (12). BRONJ occurs most commonly following tooth extraction (13,14). Indeed, it was estimated that 108 of 263 cases (41.1%) were associated with tooth extraction (6) and our own study contains a large number of patients that developed BRONJ after tooth extraction, including eight that were refractory for BRONJ. According to AAOMS guidelines, it is recommended to withdraw BP medications for three months or more from any patient requiring invasive surgery (e.g. tooth extraction) that has been on such medication for 3 years or more. However, this ‘washout’ period for BPs differs for patients with complications such as diabetes and for those taking steroids. Currently, if patients have additional risk factors, there are no clear criteria for withdrawal of BPs, and the washout period chosen is at the discretion of the attending physician. In our department, a refractory case with a history of oral alendronate for seven months developed BRONJ even after three months of withdrawal, following a tooth extraction. That patient exhibited mild periodontal disease as a risk factor, but no specific systemic risk factors. In another case of BRONJ, we identified a denture-related injury but the onset of BRONJ was otherwise spontaneous and idiopathic. Several risk factors have been proposed for BRONJ (15), although many of these are contentious. We found no significant link between responsiveness to treatment and the existence of diabetes or the use of steroids, tobacco or alcohol. However, patients with poor oral hygiene were more frequently refractory to treatment and we therefore emphasized the importance of oral hygiene, including scaling, from the outset of BRONJ treatment.

According to the AAOMS, a combination of anticancer drugs is also a risk factor in BRONJ. In our study, 28% of the refractory cases were found to have taken combined anticancer drugs so, if a combination of anticancer drugs is noted when performing conservative treatment, we suggest that the cure rate may be diminished. It has also been reported that the incidence of BRONJ in patients treated using BPs for 4–12 months was 1.5%, compared with 7.7% for those treated for 37–48 months (15-18), suggesting that the risk of BRONJ onset increases according to the duration of BP administration. However, our results demonstrate that the period of BP administration has no significant bearing on treatment responsiveness. Additionally, it has been reported that the relationship between the type of BP and the incidence of BRONJ is not clear. In our department, second generation BPs such as alendronate accounted for half of all BRONJ patients (stage 0–3). BRONJ patients accounted for less than 1 per 100,000 person-years in a European osteoporosis working group study (19), but it has also been reported that there is a high rate (4%) of BRONJ when BPs are administered orally (20).

In any case, the number of patients taking oral BPs has increased and, as a result, we must be cognizant that the number of BRONJ cases will consequently also increase, even though the incidence rate is less than that for BP injection. In this study, alendronate accounted for 33% of refractory cases, suggesting that a large number of BRONJ cases induced by oral BPs are resistant to conservative treatment. Additionally, 80% of all patients treated using zoledronate showed resistance to conservative treatment, so it was concluded that injection of BPs has a high BRONJ incidence rate compared with oral medication and that the duration of conservative treatment is likely to be longer for resistant cases.

Regarding the reasons for BP administration, 37 cases of BRONJ arose in patients treated for osteoporosis (71%). However, among refractory cases, 50% were treated using BPs for cancer bone metastasis. We conclude that the use of combination chemotherapy is a prognostic factor in the treatment of bisphosphonate-related osteonecrosis of the jaw, and thus recommend that it is necessary to carefully consider potential resistance to conservative treatment when planning the use of conservative therapy in patients receiving combination chemotherapy. It may also be necessary to consider aggressive surgical treatment in preference to conservative treatment in order to maintain QOL and shorten the healing period.

It was previously reported that serum C-terminal telopeptide cross-linked type I collagen was reduced in patients diagnosed with BRONJ, and that, in this patient group, invasive dental surgery contributes to an increased risk of BRONJ (21). Urinary NTX is effective in monitoring the effect of BPs and bone metabolism status (22) and we suggest that it may also be a predictive marker for cases resistant to conservative treatment. Additionally, in those resistant cases where urinary NTX was high, interpretation was complicated by the existence of one or more other risk factors, such as the use of combination chemotherapy. Therefore, while NTX might be useful in predicting the convalescence period, it should not be used in isolation to make prognostic decisions. Where conservative treatment fails, aggressive surgical treatment can be applied as an alternative with good results (23), although some reports suggest that full coverage with soft tissue and the use of a wound closure approach are necessary if surgical treatment is performed (24). The decision regarding the area of bone to be resected in cases of BRONJ is also controversial; it has been considered by some to be necessary to cut bone to admit a fluorescent moiety detectable by ultraviolet irradiation after the administration of doxycycline, which reportedly incorporates into new bone (25). In light of our current results, cases that are expected to be resistant to the conserva-tive treatment recommended by the AAOMS should be considered for more aggressive surgical intervention with careful consideration of the clinical features. However, further research is required to identify more prognostic markers such as NTX that could facilitate the identification of those cases that are likely to be most resistant to conservative treatment.
